# SPathDB: a comprehensive database of spatial pathway activity atlas

**DOI:** 10.1093/nar/gkae1041

**Published:** 2024-11-15

**Authors:** Feng Li, Xinyu Song, Wenli Fan, Liying Pei, Jiaqi Liu, Rui Zhao, Yifang Zhang, Mengyue Li, Kaiyue Song, Yu Sun, Chunlong Zhang, Yunpeng Zhang, Yanjun Xu

**Affiliations:** College of Bioinformatics Science and Technology, Harbin Medical University, No.157 Baojian Road, Harbin 150081, China; College of Bioinformatics Science and Technology, Harbin Medical University, No.157 Baojian Road, Harbin 150081, China; College of Bioinformatics Science and Technology, Harbin Medical University, No.157 Baojian Road, Harbin 150081, China; College of Bioinformatics Science and Technology, Harbin Medical University, No.157 Baojian Road, Harbin 150081, China; College of Bioinformatics Science and Technology, Harbin Medical University, No.157 Baojian Road, Harbin 150081, China; College of Bioinformatics Science and Technology, Harbin Medical University, No.157 Baojian Road, Harbin 150081, China; College of Bioinformatics Science and Technology, Harbin Medical University, No.157 Baojian Road, Harbin 150081, China; College of Bioinformatics Science and Technology, Harbin Medical University, No.157 Baojian Road, Harbin 150081, China; College of Bioinformatics Science and Technology, Harbin Medical University, No.157 Baojian Road, Harbin 150081, China; College of Bioinformatics Science and Technology, Harbin Medical University, No.157 Baojian Road, Harbin 150081, China; College of Bioinformatics Science and Technology, Harbin Medical University, No.157 Baojian Road, Harbin 150081, China; College of Bioinformatics Science and Technology, Harbin Medical University, No.157 Baojian Road, Harbin 150081, China; College of Bioinformatics Science and Technology, Harbin Medical University, No.157 Baojian Road, Harbin 150081, China

## Abstract

Spatial transcriptomics sequencing technology deepens our understanding of the diversity of cell behaviors, fates and states within complex tissue, which is often determined by the fine-tuning of regulatory network functional activities. Therefore, characterizing the functional activity within tissue space is helpful for revealing the functional features that drive spatial heterogeneity, and understanding complex biological processes. Here, we describe a database, SPathDB (http://bio-bigdata.hrbmu.edu.cn/SPathDB/), which aims to dissect the pathway-mediated multidimensional spatial heterogeneity in the context of functional activity. We manually curated spatial transcriptomics datasets and biological pathways from public data resources. SPathDB consists of 1689 868 spatial spots of 695 slices from 84 spatial transcriptome datasets of human and mouse, which involves 36 tissues, and also diseases such as cancer, and provides interactive analysis and visualization of the functional activities of 114 998 pathways across these spatial spots. SPathDB provides five flexible interfaces to retrieve and analyze pathways with highly variable functional activity across spatial spots, the distribution of pathway functional activities along pseudo-space axis, pathway-mediated spatial intercellular communications and the associations between spatial pathway functional activity and the occurrence of cell types. SPathDB will serve as a foundational resource for identifying functional features and elucidating underlying mechanisms of spatial heterogeneity.

## Graphical Abstract

**Graphical Abstract:**
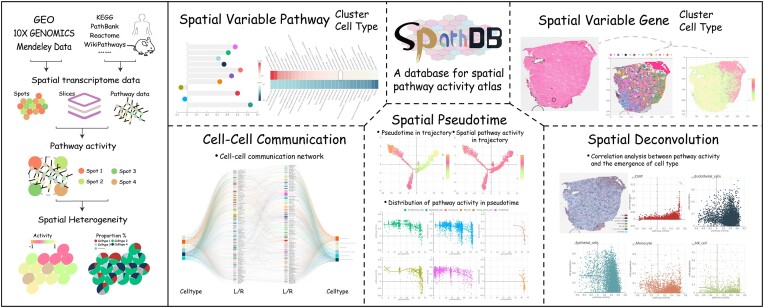

## Introduction

Spatial transcriptomics technology quantifies the molecular features of individual cells/spots while preserving the physical positional information of each cell/spot in the tissue (1,2). It is of great significance for the in-depth understanding of cellular behaviors, tissue complexity, disease occurrence and drug resistance, which are driven by the fine-tuning of regulatory network functional activities. The genes of pathways constitute important functional regulatory networks, and pathway activity analysis can provide a more powerful approach to dissecting and interpreting cellular and spatial heterogeneity (3,4). Therefore, it is necessary to investigate pathway functional activities at spatial resolution.

Spatial transcriptomics sequencing technology is significant for elucidating gene expression, functional activity and cellular interactions in spatial dimensions, thereby enhancing our understanding the mechanisms of tissue and organ development, disease occurrence, tumorigenesis and drug resistance (5,6). Starfysh integrates spatial transcriptomics data to characterize tissue-specific cell states and reveal heterogeneity (7). Analysis of spatial and single-cell transcriptomics data in human pancreatic cancer revealed cancer cell populations associated with poor prognosis (8). Cang *et al.* captured the locally high-active signaling pathways and the signaling direction of cell–cell communication at spatial locations during skin development (9). Hwang *et al.* identified multicellular dynamics associated with neoadjuvant therapy based on spatial transcriptomic data of pancreatic cancer (10). With the continuous accumulation of spatial transcriptomics data, researchers have currently developed many spatial omics databases motivated by research requirements. For example, CROST (11) and STOmicsDB (12) store manually curated spatial transcriptomics data and also provide downstream analysis and visualization. SORC (13) specifically provides visualization and analysis of spatial transcriptomics data for cancer. The SODB (14) database stores datasets from various spatial omics technologies and provides interactive analysis modules. These databases are helpful for the analysis of spatial omics data. While, the diversity of behaviors and states exhibited by cells in the spatial tissue architecture are primarily determined by the regulatory interactions among genes and the functional activities they collectively perform. Thus, a database dedicated to characterize the complex cell spatial function landscape will be important for revealing spatial heterogeneity and elucidating the underlying biological mechanisms.

Here, we developed SPathDB (http://bio-bigdata.hrbmu.edu.cn/SPathDB/), a comprehensive database for dissecting the pathway-mediated multidimensional spatial heterogeneity in the context of functional activity. The current release of SPathDB database contains following elements: (i) total 1689 868 spatial spots of 695 slices from 84 spatial transcriptome datasets of human and mouse, which involve 36 tissues (a portion of these slices was taken from 30 cancer types/subtypes); (ii) total 114 998 biological functional pathways; (iii) spatial variation pathway (SVP) exhibiting diverse functional activities across spatial slice and clusters of distinct spots populations; and (iv) trajectory distribution of spatial spots and pathway activity. SPathDB provides user-friendly interface to explore the functional activities of 114 998 pathways across spatial spots, and also provides five flexible tools to retrieve and explore gene expression and pathway activity across spatial distribution, spatial pathway functional activity along the pseudo-time trajectory, pathway-mediated spatial cell–cell communication and correlation between spatial pathway activity and cell type emergence, thereby uncovering complex relationships. In summary, SPathDB will provide important insights for investigating the driving functional features contributing to spatial heterogeneity, and serve as a comprehensive database for the identification of pathway activation signatures applying for spatial heterogeneity analysis.

## Materials and methods

### Data collection and processing

#### Data collection

##### Collection of spatial transcriptome data

Spatial transcriptome datasets of human and mouse were collected from the Gene Expression Omnibus (GEO) database (15), 10× Genomics (https://www.10xgenomics.com/), Mendeley Data (https://data.mendeley.com/) and CROST (https://ngdc.cncb.ac.cn/crost/). We retained spatial transcriptome data that include spatial location coordinates, expression profiles and histological images. Spatial tissue images can intuitively display the overall actual image of tissue sections, which allow for the comparison and correction of spatial visualization images of spots based on coordinate restoration, as well as to compare and analyze the tissue images with molecular characteristics such as pathway activity and gene expression at different spatial locations. To ensure the reliability of data and further facilitate the usage of database, only data with histological images information are retained when collecting spatial transcriptome data. Additionally, we also collected literatures related with spatial transcriptomics from PubMed (https://pubmed.ncbi.nlm.nih.gov/) to extract spatial transcriptome data containing the aforementioned information.

##### Collection of single-cell RNA sequencing datasets

We first searched for single-cell expression data from the same batch sequencing of each spatial transcriptomics dataset in the GEO database (15). If some spatial transcriptomics datasets do not have corresponding single-cell RNA sequencing (scRNA-seq) data from the same batch, we search for available scRNA-seq datasets in the GEO (15) and TISCH (16) databases based on the corresponding species, tissue or cancer type to match with the spatial transcriptomics data.

##### Collection of biological pathway data

We utilized the R package graphite (17) to obtain biological pathway data for humans and mouse. We acquired a total of 100 364 human biological pathways from seven pathway databases: KEGG (18), Panther (19), PathBank (20), PharmGKB (21), Reactome (22), SMPDB (23) and WikiPathways (24). Additionally, we obtained 14 634 mouse biological pathways from four pathway databases: KEGG (18), PathBank (20), Reactome (22) and WikiPathways (24), and extracted the interaction networks among genes within each pathway.

### Processing and clustering spatial transcriptome and scRNA-seq data

The dimensionality reduction and clustering of spatial spots were performed for each slice individually. For the expression data of each spatial slice, we used the ‘NormalizeData’ and ‘ScaleData’ functions from the Seurat R package (25) to normalize the spatial transcriptome expression profiles. We then performed principal component analysis (PCA) for dimensionality reduction. During the clustering process, selecting different resolution parameters will result in a different number of clusters. To determine the optimal resolution for clustering, we set different resolution values in randomly selected slices for clustering, and evaluate the consistency of clustering results obtained from different resolutions (see ‘Supplementary method’ section in Supplementary Data). We also explore the number of clusters that changed for different resolution values. We found that the consistency of clustering is relatively high and the number of clusters that obtained is moderate when the resolution value was set at 1.1 (Supplementary Figures S1–S2). Then, we used the ‘FindNeighbors’ (dims = 1:50) and ‘FindClusters’ (resolution = 1.1) functions to cluster the spatial transcriptome data. Finally, we employed the ‘FindAllMarkers’ function to identify marker genes for each cluster, selecting the top 10 genes based on avg_log2FC as the feature genes for each cluster.

For the collected scRNA-seq datasets, we used the Seurat R package (25) to filter out genes that were expressed in <3 cells and to exclude cells with <500 expressed genes. We then merged the samples according to their tissue types and standardized the expression profiles using the ‘NormalizeData’ function. Then, the dimensionality reduction and clustering of scRNA-seq data were performed. Marker genes for each cluster were identified using the ‘FindAllMarkers’ function.

### Annotation of cell types in scRNA-seq data

We combined two automatic methods and manual annotation strategy to annotate cell types. For each scRNA-seq dataset, the detailed steps are as follows: (i) Automatic annotation was performed using both scMayoMap (26) and SingleR (https://doi.org/doi:10.18129/B9.bioc.SingleR) methods. The scMayoMap accurately identifies and annotates cell types across different tissue backgrounds. We used the standard clustering results from upstream analysis as input, selected specific tissue type and annotated the corresponding cell types for each cluster based on the cell markers in scMayoMap (26). For SingleR, we extracted the expression matrix using the ‘GetAssayData’ function, selected appropriate reference datasets and annotated cell types based on known cell type labels from the reference datasets using the ‘SingleR’ function. (ii) The consistency of the results from the two automatic annotations was manually reviewed. The consistent annotation results from both methods were adopted. For clusters with differing annotation results, we collected cell type marker genes from the CellMarker 2.0 database (27) and combined these marker genes with the feature genes obtained from upstream analysis to manually correct the automatic annotation results. (iii) For datasets that could only be automatically annotated using SingleR (a few datasets did not have corresponding tissue types in scMayoMap), we further reviewed and corrected the results based on manual annotation. After the above steps, the ‘infercnv’ R package (https://github.com/broadinstitute/infercnv) was used to further distinguish malignant cells. All immune cells in each dataset were used as reference normal cells. Subsequently, we calculated the inferred copy number variation (CNV) score for the cells (28). We then compared the inferred CNV scores of cells in each cluster with those of the reference cells. Clusters of cells that had significantly higher inferred CNV scores compared with the reference cells (Wilcoxon rank-sum test *P*-values <0.05) were defined as malignant cells.

### Spatial pathway activity

We used the R package Gene Set Variation Analysis (GSVA) (29) to evaluate the activity of biological pathways in each spot of the spatial transcriptomics data. Based on the spatial gene expression profile corresponding to each tissue section, we constructed a spatial pathway activity profile. We normalized the count expression profiles using ‘Poisson’ distribution parameters and employed the ‘GSVA’ method to assess pathway activity across spots.

### Clustering spatial spots based on pathway activity

To reveal the spatial heterogeneity of pathway activity, we performed unsupervised clustering of the spots in the spatial transcriptomics data based on the pathway activity profile for each section. Dimensionality reduction was conducted using PCA. We then utilized ‘FindNeighbors’ and ‘FindClusters’ to cluster the pathway activity profiles, and employed ‘FindAllMarkers’ to identify differentially active pathways between the clusters. The top 10 pathways for each cluster were selected as feature active pathways.

### Spatial cell-type deconvolution

We used single-cell expression profiles from the same tissue type or cancer type as a reference, removing cell types from the reference single-cell data that had <25 cells. Subsequently, we employed the run.RCTD function from the R packages spacexr (30) and STdeconvolve (31) to perform deconvolution analysis on the spatial spots, and obtain the proportional distribution of different cell types within each spot.

We also refer to the previous methods (32,33), further based on the results of spatial spot deconvolution, to identify spots enriched with specific cell types, and the corresponding cell type label was assigned to these spots, so as to facilitate the analysis of the molecular characteristics of spots enriched with different cell types on the spatial tissue sections. Assuming that spot A has the highest proportion of cell type C_1_ with a ratio of *R*_max_ and the second-highest proportion of cell type C_2_ with a ratio of *R*. If *R*_max_ ≥ 0.5 and *R*_max_*/R ≥*2, then define spot A as a cell type C_1_ enriched spot and assign the cell type label C_1_ to spot A; otherwise, spot A is considered a mixed cell-type spot, and assign it the cell type label ‘mixed’.

### Pseudo-time analysis

To explore the functional activity and state transitions of spatial spots, we utilized the Monocle 2 R package (34) to construct pseudo-time trajectories for the spatial spots based on the spatial transcriptome profiles for each section. Additionally, combined with the cell-type labels of the spatial spots that obtained based on the results of spatial cell-type deconvolution, we separately constructed pseudo-time trajectories for the spots corresponding to different cell types.

### Cell–cell communications

To explore the communications between spatial cell types, we utilized the ‘FindLR’ function from the iTALK R package (https://github.com/Coolgenome/iTALK) to construct a receptor-ligand-mediated interaction map between cell types for each spatial section.

### Spatial variable pathway

Identifying pathways with high variability in activity across different spatial spots can better reveal and characterize the heterogeneity of functional states in spatial regions. Therefore, we identified spatially variable pathways based on the pathway activity profiles of the spatial spots. First, we standardized the pathway activity profile corresponding to each spatial section, as detailed below:


\begin{eqnarray*}act_{i,j}^t = \frac{{ac{t_{i,j}} - ac{t_{{\rm min}}}}}{{ac{t_{{\rm max}}} - ac{t_{{\rm min}}}}}\end{eqnarray*}

\begin{eqnarray*}act_{i,j}^{\prime} = {e^{act_{i,j}^t}}.\end{eqnarray*}

Here, $ac{t_{{\rm max}}}$ and $ac{t_{{\rm min}}}$ represent the maximum and minimum values on the pathway activity profile, respectively; $ac{t_{i,j}}$ denotes the activity value of pathway *i* in spot *j*; and $act_{i,j}^{\prime}$ refers to the standardized activity value of pathway *i* in spot *j*.

Then, we used the ‘FindSpatiallyVariableFeatures’ function from the Seurat R package (25) to identify spatially variable pathways (selection.method=‘moransi’) and ranked the pathways according to the degree of spatial variability in activity based on moransi.spatially.variable.rank. Spatially variable pathways were identified based on the thresholds MoransI_observed > 0.1 and MoransI_p.value < 0.05.

### Spatial variable gene

We used the ‘FindSpatiallyVariableFeatures’ function from the Seurat R package (25) to identify genes with spatial expression variability (selection.method=‘moransi’) and ranked the genes according to the degree of spatial variability in expression based on moransi.spatially.variable.rank. Genes with spatial variability were identified based on the thresholds MoransI_observed > 0.1 and MoransI_p.value < 0.05.

### Database construction

SPathDB is freely available at http://bio-bigdata.hrbmu.edu.cn/SPathDB/. The SPathDB database was developed using Java Server Pages of the Tomcat software (v6) on a Linux server. The MySQL (v5.6) data server was used to manage the datasets. Java script packages, including jQuery, Datatables and ECharts, were implemented for result data creation and visualization. All data processing and statistical analyses were performed using the R software (v4.1.0).

## Results

### Database contents and usage

#### Overview of SPathDB

**Figure 1.**
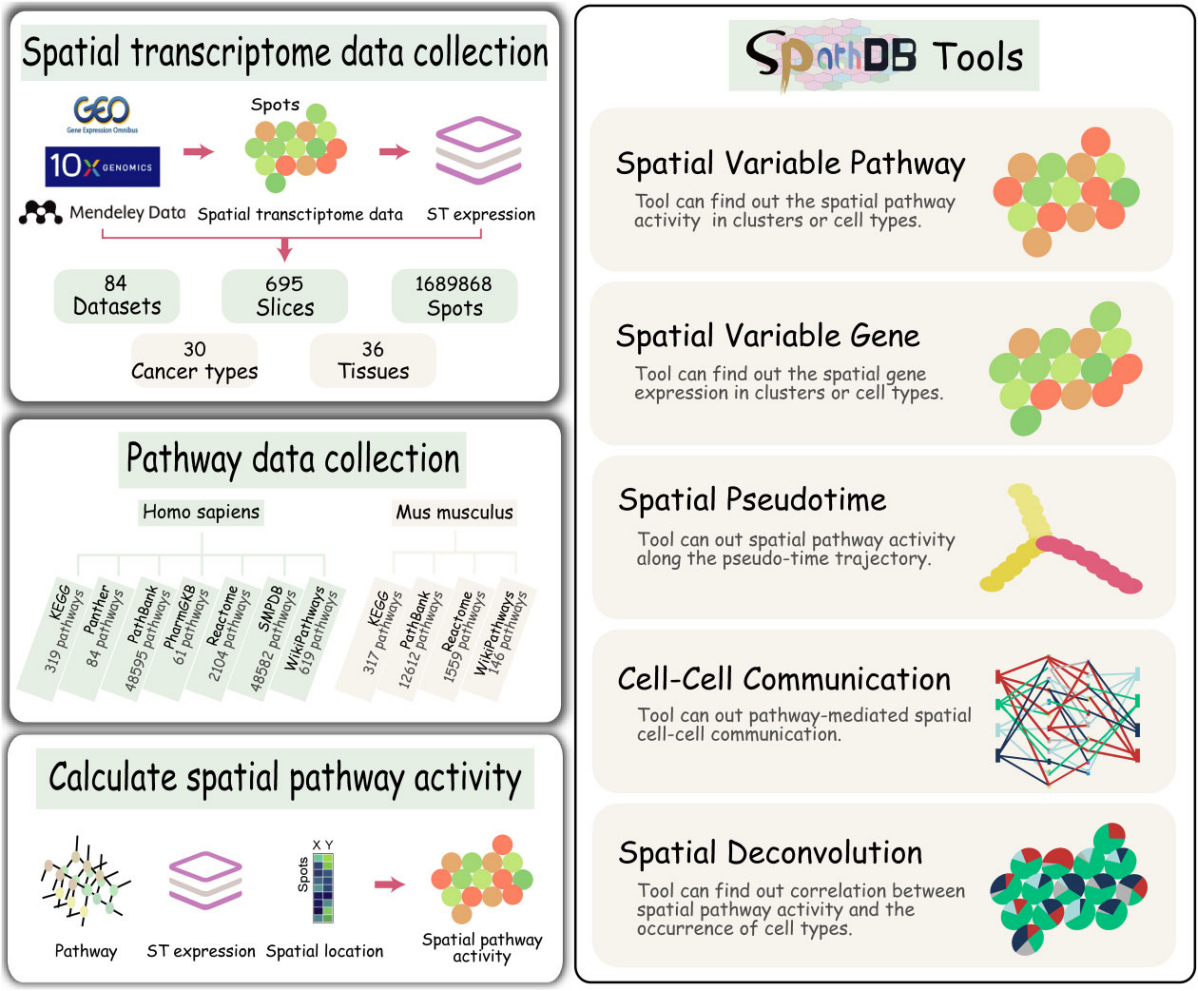
Overview of data content and functions of SPathDB. The left panel contains the database content, which includes the spatial transcriptome dataset and pathway data content, and construction of spatial pathway activity profiles. The right panel contains the tools of SPathDB to retrieve, analyze and visualize spatial pathway activity.

The design and construction of SPathDB was illustrated in Figure 1. The current version of SPathDB database contains 1689 868 spatial spots of 695 slices (sections) from 84 spatial transcriptome datasets across human and mouse, and 36 tissues. In addition, these slices are partially derived from 30 different types/subtypes of cancer. After quality control, the average number of spots per spatial slice is 2431. SPathDB performed the ‘GSVA’ method to evaluate functional activity of pathways across spatial spots of these slices. SPathDB also stores spatial functional activity for 114 998 pathways, including 100 364 human pathways and 14 634 mouse pathways. These slices of SPathDB include gene expression, pathway activities and spatial location of spots as well as the image files. In addition, as an important complement to the database, SPathDB also provides several flexible interfaces to facilitate data retrieval and analysis (Figure 1). The tools SVP and spatial variant gene (SVG) allow users to explore the functional activity distribution of pathways with spatial variation across spatial spots, clusters and cell types. Spatial pseudotime provides spatial pathway activity along the pseudo-time trajectory, cell–cell communication allows users to explore spatial active pathway-medicated cell interactions, and spatial deconvolution provides distribution of proportion of cell types across spatial spots and spatial correlation analysis between pathway activity and the emergence of cell type.

### User interface

#### Data search and browse

**Figure 2.**
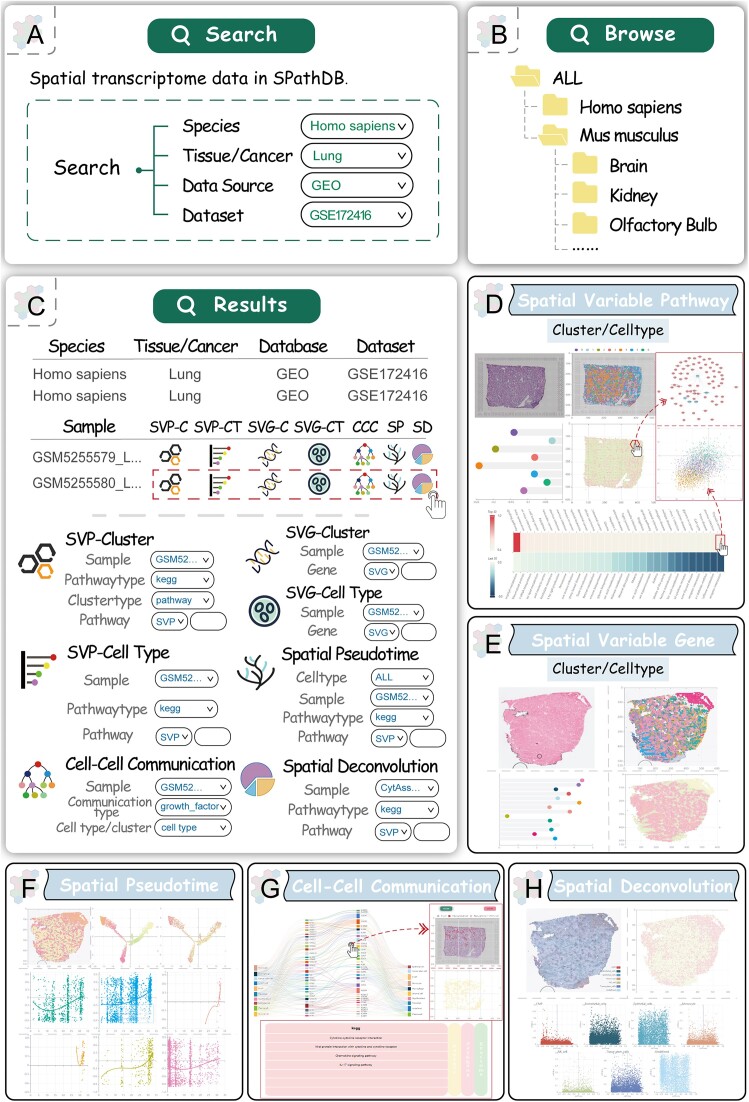
Features and utilities of SPathDB. (**A**) The search interface on the home page of SPathDB. (**B**) The browse page of SPathDB. (**C**) The result data table of search and browse, and the input of the linking analysis tools. (**D**–**H**) Five user-friendly analysis tools in SPathDB.

On the ‘HOME’ page, SPathDB provides ‘search’ interface for users to explore spatial functional activity of pathways, users need to input the species, tissue, data source and the dataset they wish to explore (Figure 2A). Users can also flexibly access all data stored in the SPathDB database through the browse page (Figure 2B). They can filter the data of interest by clicking on entries at different levels of the browsing tree, including specific species, tissue, data source and dataset. As a result of the ‘search’ and ‘browse’ page, SPathDB returns a data table in which each row provides a description of related spatial slice, the description in terms of species, tissue, data source, dataset and slice name, etc. (Figure 2C). On the results page, users can further filter the result entries by conducting keyword search. For each spatial slice, SPathDB provides multifeature interfaces for further analysis.

#### Exploration of SVP

SVPs refer to pathways that exhibit significant variation in functional activity across different spatial locations within spatial slice. Focusing on SVPs aids in understanding the differences and heterogeneity in functional states across different spatial sites or tissue neighborhoods. The SVP tool provides two sub-function pages, including SVP-Cluster and SVP-Cell Type. In these two sub-function pages, by selecting the spatial slice and pathway of interest, users can explore the distribution of SVP activity in the clusters and cell types of spatial spots (spots that enriched by specific cell type), respectively (Figure 2D). SVP-Cluster also offers selection of clusters derived from gene-based and pathway-based clustering, respectively. In addition, on the pathway activity distribution map in the result page of SVP-Cluster and SVP-Cell Type, clicking on single spot enables the visualization of the interaction network among genes within the pathway. The color of the gene nodes in the interaction network reflects the expression level of gene in the spatial spot. At the bottom of the result page, SPathDB provides a visual heatmap to display the top 30 pathways that are positively and negatively correlated with the spatial activity of the interest pathway (Figure 2D), which helps further explore the functional crosstalk and synergy in spatial regions. Furthermore, clicking on the heatmap allows for viewing a scatter plot of the spatial activity correlation between these corresponding pathways.

#### Exploration of SVG

SPathDB also provides the SVG tool, which contains SVG-Cluster and SVG-Cell Type sub-function pages that enable users to explore the spatial distribution of expression for SVG, and also the distribution of gene expression in the clusters and cell types of spatial spots, respectively (Figure 2E). In addition, the SPathDB database also provides pathway annotation information for input gene.

#### Spatial pathway activity along the pseudo-time trajectory

Trajectory analysis helps to explain the differentiation correlation of spatial cells (35), identify cell subpopulations driving disease progression and associated active pathways (36). He *et al.* (7) inferred trajectory (pseudo-space axis) based on spatial transcriptomic data and revealed the trajectory is associated with pathway gene expression. SPathDB provides Spatial Pseudotime tool for users to explore spatial pathway activity along the pseudo-time trajectory (Figure 2F). After inputting the spatial slice and pathway of interest, Spatial Pseudotime constructs developmental trajectory for spatial spots and illustrates the pseudo-time at spatial locations as well as the distribution of pathway activity along the trajectory.

#### Pathway-mediated spatial cell–cell communication

Cell–cell communication often drives heterogeneity and transitions in cellular states (37). SPathDB offers the Cell–Cell Communication tool that allows users to explore communications between different cell types on spatial slice and identify key active pathways that mediate cell communication in the spatial context (Figure 2G). After user inputs the spatial tissue slice and the type of cell communication, the Cell–Cell Communication tool visualizes detailed receptor-ligand-mediated communication between different cell types. By clicking a specific receptor-ligand connection curve on the visualization map, users can obtain the spatial distribution of the corresponding receptor/ligand expressions, the spatial distributions of different cell types and pathway annotated information of the corresponding receptor/ligand.

#### Correlation between spatial pathway activity and the occurrence of cell types

The functional activity of regulatory networks in tissue cells can drive the identity and state of cells. To better characterize the functions and heterogeneity of spatial regions, the Spatial Deconvolution tool in SPathDB allows for the deconvolution of spatial spots. Users can visualize higher resolution spatial distributions of cell types and pathway activities, and analyze the correlations between pathway activity and the occurrence of cell types in the spatial context (Figure 2H). This enables to further combine the cell types with a high proportion in spatial regions and their positively correlated highly active pathways to explore spatial heterogeneity and the functions of spatial regions, aiding in the identification of active pathway features that drive spatial heterogeneity.

#### PAI module

The personalized analysis interface (PAI) module enables users to analyze their spatial transcriptomics data based on SPathDB without requiring programming skills, allowing for personalized analysis of spatial transcriptome data. When using the online analysis tool, users should follow three steps:

1. ‘Upload file’: The user needs to click the ‘Choose File’ button in the input box to upload a data file that meets the specified format.
2. ‘Email upload’: Enter the user’s email address in the input box. After uploading the data, click the ‘Upload’ button so that SPathDB can promptly notify the user via email about the task ID.
3. ‘Job ID’: After processing is complete, the user can enter the task ID from the email notification in this input box and click the ‘Search’ button to view the analysis results of the uploaded data.

By following these three simple steps, users can conveniently perform spatial transcriptomics analysis and obtain the desired results.

The Spatial Deconvolution PAI, upon inputting data, leverages spatial transcriptomics data to provide an in-depth understanding of the composition of cell types within spots and their relationship with pathway activity. Through deconvolution analysis, the results offer the distribution of different cell types within each spot, a spatial representation of pathway activity and correlation analysis between pathway activity and the emergence of cell types. This aids users in further exploring potential regulatory mechanisms. File preparation is as follows:

- Spatial transcriptomics raw file (.h5);
- Spatial location information file (.zip);
- Single-cell expression profile file (.txt);
- Single-cell annotation file (.txt); and
- Gene set file for biological pathway (.gmt).

The Spatial Pseudotime PAI visualizes the pseudo-time trajectories of spots, revealing the dynamic activity of cells within spots during development and their relationship with pathway activity. Users can intuitively observe the spatial distribution of cell types and the dynamic changes of pathway activity along pseudo-time trajectories. This aids in understanding the spatiotemporal relationship between cell types and pathway activity within tissue section. File preparation is as follows:

- Spatial transcriptomics raw file (.h5);
- Spatial location information file (.zip);
- Spatial transcriptomics expression profile annotation file (.csv); and
- Gene set file for biological pathway (.gmt).

The Cell–Cell Communication PAI provides an intuitive and interactive platform. It visually displays the communication relationships between cell types through ligand-receptor pairs using Sankey diagrams, helping users better understand cell interactions within tissues. File preparation is as follows:

- Spatial transcriptomics raw file (.h5);
- Spatial location information file (.zip); and
- Spatial transcriptomics expression profile annotation file (.csv)

### Case study

**Figure 3.**
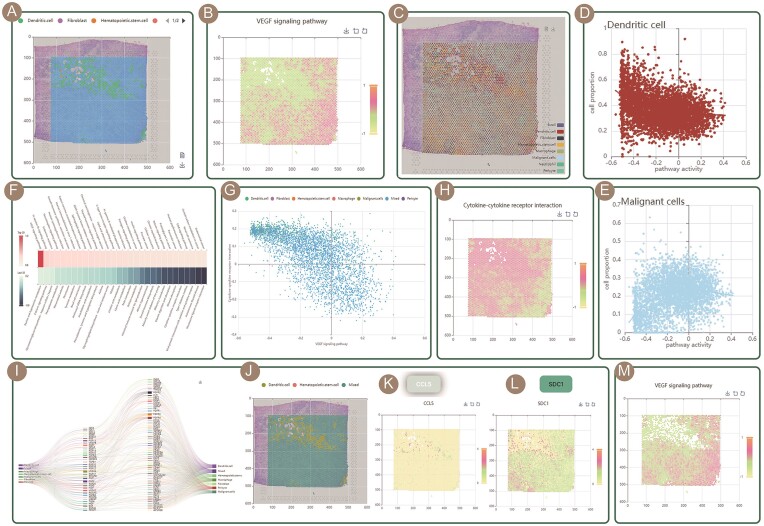
Example application of SPathDB. (**A**) The spatial distribution of cell types across spots. (**B**) The spatial distribution of activity of VEGF signaling pathway. (**C**) Distribution of different cell types across spatial spots based on deconvolution. (**D**–**E**) Scatter plots showed the correlation between the activity of VEGF signaling pathway with dendritic cells and malignant cells, respectively. (**F**–**G**) The spatial correlation heatmap and scatter plot of activities between VEGF signaling pathway and cytokine–cytokine receptor interaction pathway. (**H**) The spatial distribution of activity of cytokine–cytokine receptor interaction pathway. (**I**) Cell–cell communication map for ‘Parent_Visium_Human_BreastCancer’ section. (**J**–**L**) Visualization of the spatial expression of ligand/receptor pair: CCL5-SDC1. (**M**) The spatial distribution of activity of VEGF signaling pathway (dendritic cell-enriched spots were hidden).

To demonstrate the potential application of SPathDB, we analyzed the spatial pathway functional heterogeneity of breast cancer tissue slices (sections). We browsed the breast cancer section ‘Parent_Visium_Human_BreastCancer’ under the directory of Homo sapiens - Breast cancer - 10X Genomics on the Browse page for the analysis. First, we focused on the pathways with spatially active variation. We utilized the SVP-Cell Type sub-function under the ‘Spatial Variable Pathway’ tool to analyze the tissue section, inputting the corresponding parameter information for the section (sample), including: Species, Tissue/Cancer, Data Source and Sample. The ‘KEGG’ was selected for ‘Pathwaytype’ option. In the SVP dropdown list under the Pathway option, multiple signaling pathways with highly variable spatial activity on the slice were identified, including the ‘MAPK signaling pathway’, ‘PI3K-Akt signaling pathway’, ‘mTOR signaling pathway’, ‘VEGF signaling pathway’, etc. These signaling pathways have been confirmed to be oncogenic pathways and are closely associated with breast cancer (38–40). Among them, the VEGF signaling pathway is associated with tumor angiogenesis (41). We further explored the activity distribution of this pathway across different cell types and found that the VEGF signaling pathway exhibited higher activity in ‘mixed’ spot region, while lower activity in dendritic cell-enriched region (Figure 3A and B). Furthermore, using the Spatial Deconvolution tool, at a higher resolution, analyze the correlation between the proportions of different cell types in the spots and the activity of the VEGF signaling pathway on the tissue slice (Parent_Visium_Human_BreastCancer). Malignant cells were found in many mixed cell-type spots (Figure 3C). Moreover, it was found that the proportion of dendritic cells (malignant cells) in spots on the tissue slices showed a negative (positive) correlation trend with the activity of VEGF signaling pathway (Figure 3D and E). These findings are consistent with the fact that malignant cells have a high functional status of angiogenesis.

Next, through the spatial correlation heatmap of pathway activity (the above result of using SVP-Cell Type sub-function under the ‘Spatial Variable Pathway’ tool), we found that the activity of the SVP cytokine–cytokine receptor interaction pathway is strongly negatively correlated (correlation coefficient: −0.68) with the VEGF signaling pathway (Figure 3F and G). We further explored the activity distribution of the cytokine–cytokine receptor interaction pathway across the spatial spots and found that its activity exhibited a mutually exclusive distribution with that of the VEGF signaling pathway. Specifically, the cytokine–cytokine receptor interaction pathway showed higher activity in the dendritic cell-enriched region, while lower activity in the ‘mixed’ spot region (malignant cell distribution region) (Figure 3H). The above results indicate that, within breast cancer tumor tissues, tumor-derived cytokines may activate the antitumor immune response of dendritic cells. This analysis has corresponding supporting evidence (42,43). Further, we used Cell–Cell Communication tool to dissect intercellular communications between dendritic cell and ‘mixed’ cell type on the spatial spots of ‘Parent_Visium_Human_BreastCancer’ section, and found that ligand/receptor pair CCL5-SDC1 mediated communications between these two cell types (Figure 3I). Click the CCL5-SDC1 connection curve on the interaction map to visualize the ligand/receptor spatial expression (Figure 3J–L), and it was found that CCL5 in the cytokine–cytokine receptor interaction pathway tends to be expressed in dendritic cell spatial region. Furthermore, we observed that the activity of the VEGF signaling pathway located in close proximity to the core spot region of dendritic cell-enriched was relatively low (Figure 3M). The above analysis results suggest the potential spatial interaction pattern between breast cancer cells and dendritic cells, as well as the spatial heterogeneity of functional activities.

## Discussion

Here, we describe a database, SPathDB (http://bio-bigdata.hrbmu.edu.cn/SPathDB/), which contains 1689 868 spatial spots of 695 slices from 84 spatial transcriptome datasets, 36 tissues and diseases, including cancer. SPathDB provides user-friendly interfaces to browse the functional activities of 114 998 pathways across spatial spots, and also provides flexible tools to retrieve, analyze and visualize data. Users can analyze spatial slices of tissues or diseases of interest based on the existing data in SPathDB to discover meaningful features such as highly active pathways in spatial regions, which can further guide the analysis for their own tissue samples. In addition, PAI is also provided in SPathDB, allowing users to upload their own tissue samples for corresponding analysis. Users can also compare the analysis results of their own tissue samples with ones of tissue slices stored in SPathDB, discovering common and unique features.

Cell identity, behavior and state are determined by the fine-tuning of functional activity of pathway regulatory networks. SPathDB calculates the correlation between the proportion of cell types and pathway activity across spatial spots (i.e. evaluates correlation between spatial pathway activity and the occurrence of cell types). Indeed, pathway activity scores represent averaged signals of all cells from various cell types within a spot. This correlation analysis does not accurately reveal cell type-specific pathway activity in spatial tissue. Evaluation of cell-type specific pathway scores and identification of highly active pathways specific to cell types will be more biologically meaningful. Thus, in future updates of SPathDB, we will consider integrating spatial transcriptome data with single-cell resolution to improve this problem.

We compared SPathDB with public databases and web-based platforms including SpatialDB (44), STOmicsDB (12), SPASCER (45), SORC (13), SCAR (46), CROST (11) and Aquila (47) for visualizing and storing spatial transcriptomics data. SPathDB offers more data volume in terms of the number of dataset, spatial spots, slices and tissue types than most of those databases except for CROST (11) and STOmicsDB (12) (Supplementary Table S1). SpathDB displays a significant advantage over all seven databases in terms of the number of biological pathways (Supplementary Table S1). SPathDB offers richer and unique features of downstream analysis tools and functionalities compared with these seven public databases (Supplementary Table S2).

For some functions shared with other databases, SPathDB also exhibits unique features. For example, the SVP detection function, which can also be implemented in the SpatialDB (44) and CROST (11) databases. This established approach, first identifying SVGs and then performing enrichment analysis to obtain spatially variable pathways, is useful for interpretation of spatial feature genes. SPathDB provides the following aspects of differs and potentially improves upon the established approach: (i) In the established approach, there need a threshold to determine SVG, and the choice of this threshold is often arbitrary. (ii) If the activity of two SVG genes within the same pathway *k* is negatively correlated across spatial spots, the average signal of these genes may lead to the unchanged pathway activity in spatial spots. SPathDB directly identifies SVPs based on spot-level pathway activities that can reduce the possibility of identification of the aforementioned pathways. (iii) The statistical *P*-values are easily influenced by the choice of the background gene set size. The method of SPathDB to detect SVPs can avoid the calculation of enrichment significance *P*-value. (iv) SPathDB evaluates pathway activity at spot level and detects SVPs, which are able to directly fetch the most relevant pathways and their highly active spots for downstream analysis. Spatial spots clustering is a common function in current spatial transcriptome databases. Except for the clustering based on gene expression, SPathDB also provides the function of clustering based on pathway activity (an option under directory of the Spatial Variable Pathway→ SVP cluster tool), which is one of the unique features for SPathDB (Supplementary Table S2). The identity and state of cells are often determined by the functional activities of gene regulatory networks. Pathway activity can directly reflect the state of cells at the functional level and can be used as a stable marker. Tissues contain cells under active state transition, which is a common situation in tumor or developmental datasets (48–51). In such cases, dimensionality reduction and clustering based on spot resolution of pathway functional activity may be more reliable.

In summary, SPathDB exhibits considerable potential to complement currently public databases and platforms for storing, analyzing and visualizing spatial transcriptomics data. We believe that SPathDB will be a functional resource for the research of spatial transcriptome, and discovery pathway activation signatures for spatial regions and spatial heterogeneity. The extensions of database will continue, including adding newly pathways and spatial omics datasets, as well as newly analysis tools in the update release.

## Supplementary Material

gkae1041_Supplemental_File

## Data Availability

SPathDB is freely available online at http://bio-bigdata.hrbmu.edu.cn/SPathDB/.
