# Supplementary material for: SPathDB: a comprehensive database of spatial pathway activity atlas
Source: Nucleic Acids Res. 2024 Nov 15;53(D1):D1205–14. doi: 10.1093/nar/gkae1041 (PMC11701687; doi:10.1093/nar/gkae1041)
Supplement: gkae1041_Supplemental_File [file gkae1041_supplemental_file.pdf]

Supplementary method

Determination of the optimal resolution for clustering

To determine the optimal resolution for clustering, we set different resolution values in randomly selected slices for clustering of spatial spots, and evaluate the consistency of clustering results obtained from different resolutions. The specific steps are as follows:

(i) A certain number (50) of slices are randomly selected. For each randomly selected slice, dimensionality reduction and clustering were performed at different resolution values (from 0.1 to 1.9, with an interval of 0.2);

(ii) For each slice's spot clustering results, calculate the consistency score between spot clustering results under two adjacent resolution settings (Jacquard coefficient value). For example, for the clustering results obtained with two resolutions (0.9 and 1.1), matrix M represents the spot-to-spot clustering matrix at resolution=0.9, where each row and column represent spot. If two spots are clustered together, the corresponding element value in matrix M is 1; otherwise, it is 0. Similarly, matrix N is obtained based on the clustering results at resolution=1.1. Then, the consistency score *JC* value for the clustering results obtained under two different resolutions is calculated as follows:

$$JC = \frac{|A \cap B|}{|A \cup B|}$$

Where, A and B are the set of position coordinates of elements with value 1 on matrix M and N, respectively.

(iii) For each selected slice data, calculate the consistency scores between the clustering results obtained with different resolution settings and their two nearest neighbor threshold values (e.g. 0.9 and 1.1 are two adjacent set resolution values, 1.1 and 1.3 are also two adjacent set resolution values).

Supplementary Figures

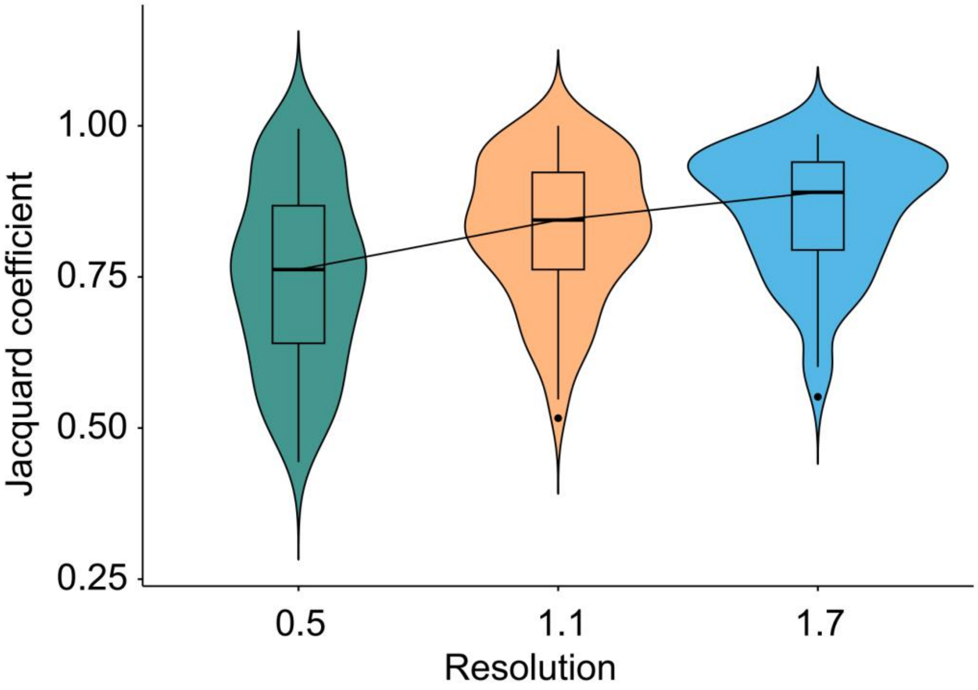

Figure S1. Consistency for clustering results between resolution setting (0.5, 1.1 and 1.7) and its two adjacent set resolutions (i.e. the results between resolution 0.5 and two adjacent set resolutions 0.3 and 0.7; 1.1 and two adjacent set resolutions 0.9 and 0.3; 1.7 and two adjacent set resolutions 1.5 and 1.9).

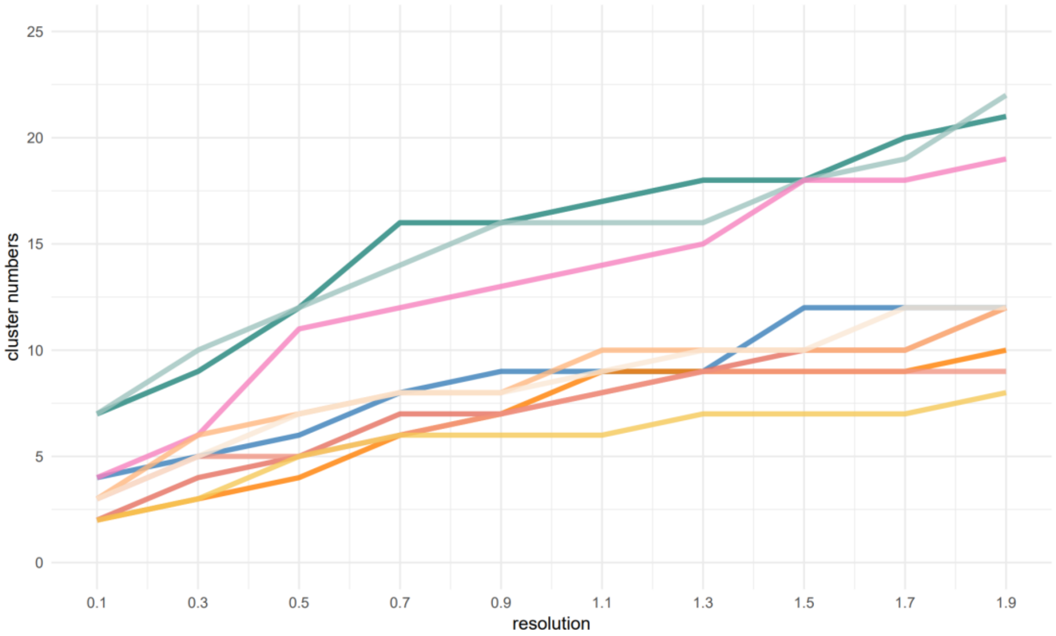

Figure S2. The line chart of the changes in the number of clusters with different resolutions. X-axis: the resolution setting for clustering, Y-axis: the number of clusters. Each line represents the results obtained based on one randomly selected spatial tissue slice.

Supplementary Tables

Table S1. Comparison of SPathDB with published spatial data resources

	Tissue	Datasets	Slices	Spots	Pathways
**SPathDB**	**36**	**84**	**695**	**1689868**	**114998**
SpatialDB	8	13	484	163777	41655
STOmicsDB	28	139	1216	7112177	41655
SPASCER	13	22	692	457057	58757
SORC	17	82	269	722899	41672
SCAR	16	13	39	124476	40939
CROST	30	150	792	1664982	41655
Aquila	20	43	963	749710	-

Table S2. Comparison of SPathDB functionality with published spatial data resources

	Spots annotate-ion/deconvolution	SVG	SVP	Spots clustering based on pathway	Spot-resolution pathway activity	Pathway topology visualization	Spatial pathway correlation analysis	Pseudo-time analysis	Cell-Cell Communi-cation	Personaliz ed analysis tools
**SPathDB**	**Yes**	**Yes**	**Yes**	**Yes**	**Yes**	**Yes**	**Yes**	**Yes**	**Yes**	**Yes**
SpatialDB	No	Yes	Yes	No	No	No	No	No	No	Yes
STOmicsDB	Yes	Yes	No	No	No	No	No	No	Yes	Yes
SPASCER	Yes	Yes	Yes	No	No	No	No	No	Yes	No
SORC	Yes	Yes	No	No	No	No	No	No	Yes	No
SCAR	Yes	Yes	No	No	No	No	No	No	Yes	No
CROST	Yes	Yes	Yes	No	No	No	No	No	Yes	Yes
Aquila	Yes	Yes	No	No	No	No	No	No	Yes	Yes
